# Assessment of Multivariate Neural Time Series by Phase Synchrony Clustering in a Time-Frequency-Topography Representation

**DOI:** 10.1155/2018/2406909

**Published:** 2018-03-21

**Authors:** M. A. Porta-Garcia, R. Valdes-Cristerna, O. Yanez-Suarez

**Affiliations:** Neuroimaging Research Laboratory, Electrical Engineering Department, Edificio T-227, Universidad Autónoma Metropolitana Iztapalapa, Av. San Rafael Atlixco 186, Col. Vicentina, Del. Iztapalapa, 09340 Ciudad de México, Mexico

## Abstract

Most EEG phase synchrony measures are of bivariate nature. Those that are multivariate focus on producing global indices of the synchronization state of the system. Thus, better descriptions of spatial and temporal local interactions are still in demand. A framework for characterization of phase synchrony relationships between multivariate neural time series is presented, applied either in a single epoch or over an intertrial assessment, relying on a proposed clustering algorithm, termed Multivariate Time Series Clustering by Phase Synchrony, which generates fuzzy clusters for each multivalued time sample and thereupon obtains hard clusters according to a circular variance threshold; such cluster modes are then depicted in Time-Frequency-Topography representations of synchrony state beyond mere global indices. EEG signals from P300 Speller sessions of four subjects were analyzed, obtaining useful insights of synchrony patterns related to the ERP and even revealing steady-state artifacts at 7.6 Hz. Further, contrast maps of Levenshtein Distance highlight synchrony differences between ERP and no-ERP epochs, mainly at delta and theta bands. The framework, which is not limited to one synchrony measure, allows observing dynamics of phase changes and interactions among channels and can be applied to analyze other cognitive states rather than ERP versus no ERP.

## 1. Introduction

There is a growing interest among the neuroscientific community to unravel the intricate neural mechanisms involved in the broad integration of different brain structures, which enable the emergence of cognitive processes. Several studies conducted with electroencephalography (EEG) and magnetoencephalography (MEG) have provided evidence that supports the idea of neural synchronization intrinsic to mental tasks, with the fluctuating disposition of communication channels in the nervous system, especially between active regions in the brain [1–5].

In this regard, phase locking analysis of neural oscillations and other different measures of synchronization has gained attention, as several methods have been developed to provide a quantitative view of synchronism in brain sources and their behavior, estimating phase synchrony (PS) from different perspectives, depending on the purpose of the study in question [6]. This same variety of methods and proposals causes lack of agreement in the terminology used to refer to all these measures. Roach and Mathalon have provided a wide review attempting to clarify this situation [7]. Thus, for the sake of following a standard of terms, descriptions of any PS measure will follow the referred publication.

In order to perform PS analysis, instantaneous phase information of EEG signals must be extracted. Most methods are based on wavelet analysis [6–10]. Another common technique besides wavelets for extracting instantaneous phase values from the analytical signal is the Hilbert transform. Analytic phase from wavelets or Hilbert transform has been shown to give almost same results as Short Time Fourier Transform adjusting the filter settings adequately [11, 12]. There are also other Time-Frequency (TF) decompositions used for obtaining phase information, such as Rihaczek distribution, Empirical Mode Decomposition, sinusoidal quadrature filters, and Matching Pursuit [13–16].

In general, for the study of PS, it can be said that there are two main approaches: phase locking and phase coherence. The former refers to the event-related phase locking across trials regarding an event's onset over one electrode, that is, the Phase Locking Factor (PLF). If instantaneous phase angles between trials are closer to a uniform distribution over the unit circle, the PLF is close to zero; otherwise, it is close to one if instantaneous phase angles between trials are highly synchronized in the same direction over the unit circle. The latter approach, phase coherence, also called Phase Locking Value (PLV), or within the context, the* event-related phase coherence across trials*, evaluates consistency of phase differences between 2 electrodes across trials, also with values between 0 and 1. As the reader already noticed, each measure determines different types of PS; therefore, both measures can be complementary to each other [7].

Other types of measures, such as linear coherence or magnitude squared coherence, are not suitable to analyze PS; unlike PLF and PLV, both measures yield results weighted by magnitude, and the interpretation of these becomes unclear, since phase synchronization patterns and amplitude changes are not necessarily related to the same neural process [6–8]; Rosenblum demonstrated that PS of chaotic oscillators is possible, where bounded phase differences exist and variations of amplitude are chaotic and uncorrelated [17]. The Phase Cross-Coherence (PCC) eliminates amplitude information and produces a function of phase differences averaged across trials [6].

All PS measures mentioned above focus on the evaluation of intertrial phase consistency over an individual EEG channel or phase differences between signals from two recoding sites, that is, providing only univariate or bivariate approaches. Nevertheless, the complete scenario involves a multichannel recording; thus a bivariate approach may not capture relevant information of all the dynamics and interactions of the full system [18, 19]. Thereupon, existing methods of multivariate synchronization analysis comprise even other metrics besides PS, based on different types of correlation measures. Correlation between probabilities of recurrence is used to measure PS, clearly distinguishing preseizure and seizure states of epileptic EEG [20, 21]. Based on Random-Matrix Theory (RMT), Osorio and Lai compute the average phase synchronization times (APSTs) among pairs of channels in order to construct a matrix, from which they use both the determinant and the eigenvalue spectra for assessing synchronization [22]. Li et al. presented another method based on RMT, using equal-time correlation instead of PS, and then the eigenvalue decomposition is used to calculate a global synchronization index that increases during epileptic seizures [23]. Mutlu et al. extend the concept of phase differences between two signals, mapping these differences onto an *N*-dimensional hyperspherical coordinate system; however, the authors later reported that Hyperspherical Phase Synchrony (HPS) is dependent on how the phase differences are sorted, which is corrected with another hyperdimensional coordinate system [19, 24]. Alba et al. proposed a visualization system with multitoposcopic graphs and Time-Frequency-Topography (TFT) maps for synchrony patterns, indicating increase, decrease, or an equal level of synchronization between pairs of electrodes with respect to a previous state, using different PS bivariate measures [15].

Some other approaches aim to improve the resolution of the TF decomposition used for extracting phase information. Aviyente and colleagues used a reduced interference distribution-Rihaczek (RID-Rihaczek) for computing PLV [25]. Subsequently, the authors extend their method to quantify all possible pairwise comparisons and analyze those interactions between electrodes through a graph clustering algorithm, which allows overlapping clusters, and each electrode has a “participation score” that reflects their significance in the formation of a cluster [26]. Previous works also conceive the idea of clustering with degrees of membership. Allefeld and Kurths addressed the multivariate synchronization as a mean-field cluster of oscillators that participate in different degrees, that is, how close an oscillator phase is close to a reference phase, which is determined by the circular mean of all oscillator phases [27]. Nevertheless, the single cluster assumption dismisses other possible cluster formations. Later, the authors made a generalization of the cluster analysis to correct this issue based on eigenvalue decomposition of a matrix containing indices of bivariate synchronization strength, associating each eigenvalue greater than one to a cluster [28]; however, the one-to-one correspondence between dominant eigenvectors and clusters is not always fulfilled [29].

Summarizing, multivariate methods help in perceiving overall synchronization patterns, providing a global index instead of matrices of bivariate comparisons [19]. Since many of these investigations focus on epilepsy studies, it makes sense to provide a general assessment of the synchronization state of the system with a crisp numerical value in order to distinguish seizure and preseizure conditions. Rather than a global index and aiming to characterize a broader variety of cognitive states, such as mental tasks for Brain-Computer Interface (BCI), the framework proposed in this article points to observing the dynamics of phase changes along multivariate neural time series over the TF plane and projecting their interactions in TFT maps.

The proposed clustering algorithm, Multivariate Time Series Clustering by Phase Synchrony (mCPS), establishes local relations by means of clusters of highly synchronized signals in each sample time, allowing exploring these phase associations through all samples searching for patterns of cluster formations. Additionally, our proposal also addresses an across-trials perspective. Thus, it can be said that the PS measure used in this work is more related to PLF (circular variance) rather than to phase coherence (consistency of phase differences), applied channel-wise. Haig et al. proposed a similar conception of PS, which lacks an automatized selection of synchronized signals via clustering [30].

Beyond yielding a PS measurement and a TFT portrayal, the framework also provides contrast maps of Levenshtein Distance (LD) as a metric for visual analysis and comparison of differences in PS patterns between different conditions (in this case, ERP and no-ERP epochs), as well as TF images of channels, highlighting which clusters of PS can be related to the changes of power due to the ERP. While some of the methods mentioned before use clustering analysis, like [26], most of them are fuzzy clusters in short time windows and without topographic representation. The way mCPS is conceived requires hard clustering, as it will be further detailed.

## 2. Materials and Methods

### 2.1. Simulated EEG and Experimental Data

Several experiments were carried out with both synthetic and real EEG signals (sEEG and rEEG, resp.) in order to determine the extent to which our framework is capable of retrieving reliable and useful information (presented as clusters of electrodes) that allows establishing relationships between highly synchronized EEG channels and the brain activity of interest through time samples and over different bandwidths. The sEEG was built based on a linear mixing model of *N*_*s*_ independent sources *S* = (*s*_1_, *s*_2_,…,*s*_*N*_*s*__)^*T*^, with a sampling frequency of 256 Hz, resulting in *N*_ch_ observed signals *C* = (*c*_1_, *c*_2_,…,*c*_*N*_ch__)^*T*^. Contributions of every *s*_*i*_  (*i* = 1,2,…, *N*_*s*_) through the *N* discrete-time samples (*n* = 1,2, 3,…, *N*) are weighted by the *N*_ch_ × *N*_*s*_ matrix *W*, which is determined by the inverse-square law of distances between *C* and *S* locations:

(1) C=WS;c1,n⋮cNch,n=w1,1⋯w1,Ns⋮⋱⋮wNch,1⋯wNch,Nss1,n⋮sNs,n.

Spatial location of each electrode *c*_*j*_  (*j* = 1,2,…, *N*_ch_) corresponds to the basic 10–20 international system [32] over a unit sphere. The volume conduction of the EEG model was assumed to be homogeneous and isotropic. The complete sEEG record is constructed with 30 epochs of 3 seconds, each of them containing a simulated Visual Evoked Potential (VEP) centered at 1.5 s from the epoch onset (peak amplitude at 1500 ms and constant across trials). Equation (2) describes the construction of the VEP:

(2) $$\begin{array}{c} VEP=\frac{1}{\sqrt{2\pi }}e^{-{\left(\left(n-\mu \right)/\sigma^{2}\right)}^{2}}\sin\left(\;\frac{2\pi fn}{N}\gamma \;\right), \end{array}$$

where *f* = 10 Hz, *μ* = 0.5*N*, *σ*^2^ = 0.125*N*, *γ* = (*N* − 0.5*n*)/*N*, and *n* = 1,2, 3,…, *N*. Besides the VEP, sources *S* comprise three different types of noise components: (a) harmonics, which vary in amplitude, frequency of the sinusoidal oscillations, and initial phase and (b) white Gaussian and (c) colored Gaussian *cgn* noise. Localization (*x*, *y*, *z*) of *S* within the brain area of the model can be either a fixed position or a linear displacement or with rotational motion.

In order to assess the framework with rEEG, four subjects (S2, S5, S6, and S7) were selected from a record of P300 evoked potentials [33] using the P300 Speller proposed by Farwell and Donchin [34] (available at http://bnci-horizon-2020.eu/database/data-sets). The subjects were patients with amyotrophic lateral sclerosis and were naive to BCI training. The authors recorded eight EEG signals according to 10-10 standard (Fz, Cz, Pz, Oz, P3, P4, PO7, and PO8) using active electrodes, referenced to the right earlobe and grounded to the left mastoid. EEG signal was digitized at 256 Hz and band-pass-filtered between 0.1 and 30 Hz. Subjects were required to spell seven predefined words of five characters each by controlling the P300 matrix speller. It should be mentioned that no extra preprocessing was performed over the data. The first three runs (15 trials in total) are described as “calibration runs” and runs 4–7 are the “testing runs” where participants were provided with feedback.

### 2.2. Clustering EEG Channels according to Circular Variance

#### 2.2.1. Extraction of Phase Information

Given *N*_ch_ (for sEEG, *N*_ch_ = 19 and, for rEEG, *N*_ch_ = 8) signals, a TF decomposition is performed over the continuous EEG with predefined bandwidths at center frequencies:

(3) $$\begin{array}{c} f_{k}=e^{\ln\left(f_{min}\right)+\left(\left(\ln\left(f_{max}\right)-\ln\left(f_{min}\right)\right)/K\right)k};\;k=1,2,3,\ldots ,K, \end{array}$$

where *f*_min_ = 1, *f*_max_ = 12 Hz, and *K* = 12 for both sEEG and rEEG. Such decomposition is carried out with a Continuous Wavelet Transform (CWT) at peak frequencies *f*_*k*_ from (3) with complex Morlet wavelets:

(4) $$\begin{array}{c} \Psi \left(\;n,f_{k}\;\right)=e^{i2\pi f_{k}t}e^{-n^{2}/2\varsigma^{2}};\;n=1,2,3,\ldots ,N, \end{array}$$

where *ς* = *ϱ*/2*πf*_*k*_ is the standard deviation of the Gaussian function used to make each Ψ and *ϱ* is the number of wavelet cycles (in this case, *ϱ* = 4). Then, the instantaneous phase is obtained from (5), using implementation of the four-quadrant inverse tangent:

(5) $$\begin{array}{c} \theta_{j}\left(\;n,f_{k}\;\right)=\arctan\left(\;\frac{imag\left(\Psi \right)}{real\left(\Psi \right)}\;\right);\;j=1,2,\ldots ,N_{ch}. \end{array}$$

#### 2.2.2. Multivariate Time Series Clustering by Phase Synchrony (mCPS)

Algorithm 1 explains how mCPS works, which is based on directional statistics to measure the degree of phase locking and formation of clusters. The circular spread in angular data can be computed with the magnitude of the so-called* mean resultant vector *$\bar{R}$ [35]. Directional data (in this case, *θ*_*j*_(*n*, *f*_*k*_) of the *N*_ch_ signals) can be observed as points *x*_*j*_ = (cos⁡*θ*_*j*_, sin⁡*θ*_*j*_) over the unit circle. Then, the Cartesian coordinates of the center of mass can be expressed as (*A*, *B*), where

(6) A=1Nch∑j=1Nchcos⁡θj;B=1Nch∑j=1Nchsin⁡θj.

Therefore, $\bar{R}=\sqrt{A^{2}+B^{2}}$. Magnitude of $\bar{R}$ is close to 1 when EEG channels are highly phase-locked; it is close to zero otherwise. Porta-Garcia et al. presented an example using magnitude changes of vector $\bar{R}$ over time in a determined group of EEG channels comparing two different conditions [36]. The functioning of mCPS over EEG channels according to circular variance is as follows.

Once *θ*_*j*_(*n*, *f*_*k*_) is retrieved for the entire EEG, the procedure CreateFuzzyClusters generates *fC* fuzzy clusters of electrodes for each time sample *n* and for each center frequency *f*_*k*_. The threshold *r*  (0 < *r* < 1) defines whether or not an electrode is assigned to a determined *fC*, and as fuzzy clusters consider intersections of cluster elements, the main task of the procedure ConvertToHardClusters is to obtain hard clusters *hC* by preserving clusters with higher value of *r* and proceed to eliminate intersections iteratively of the remaining *fC* in such a way that *hC*_1_∩*hC*_2_∩⋯∩*hC*_*i*_ = *∅*; *i*∣1 < = *i* < = *N*_ch_. Therefore, the result of mCPS is a new *N*_ch_ × *N* matrix cEEG, containing the cluster labels to which each EEG channel belongs in each time sample *n*.

#### 2.2.3. Cluster Labeling

**Figure 1:**
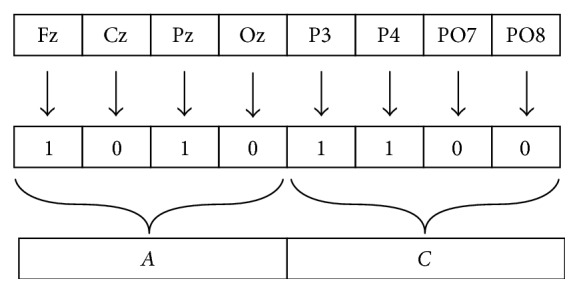
Example of hexadecimal cluster labeling for an 8-channel EEG array, where *AC* represents the cluster containing Fz, Pz, P3, and P4.

Every run of mCPS is bounded for each time sample *n*, and an arbitrary numeric label is assigned to each cluster. Then, an example of generated clusters could be *hC*_1_ = {P3, P4, O*z*} for *n* = 1 and *hC*_2_ = {P3, P4, Oz} for *n* = 2. In this case, numeric labels 1 and 2 do not provide any useful information of cluster content. In order to establish a meaningful relationship that reflects that *hC*_1_ and *hC*_2_ are actually the same cluster, a labeling system was developed based on hexadecimal words that encode which electrode belongs to the cluster and then assign a specific color in a one-to-one relationship to represent clusters in a TFT map, which will be described further. In Figure 1, it can be observed that each hexadecimal digit corresponds to binary bits of electrode quartets, where digit 1 means that the electrode is assigned to a determined cluster if magnitude of $\bar{R}$ is greater than threshold *r*. Therefore, a hexadecimal word of two digits encodes the cluster membership for eight EEG channels. As a consequence of this encoding system, a slightly different hue of color label should depict similarity between clusters, for example, a blue cluster containing electrodes P3, Pz, and P4 and a lighter blue cluster that only contains P3 and Pz. Hence, the matrix cEEG now has as elements the cluster labels of hexadecimal words.

#### 2.2.4. Construction of Time-Frequency-Topography (TFT) Maps

**Figure 2:**
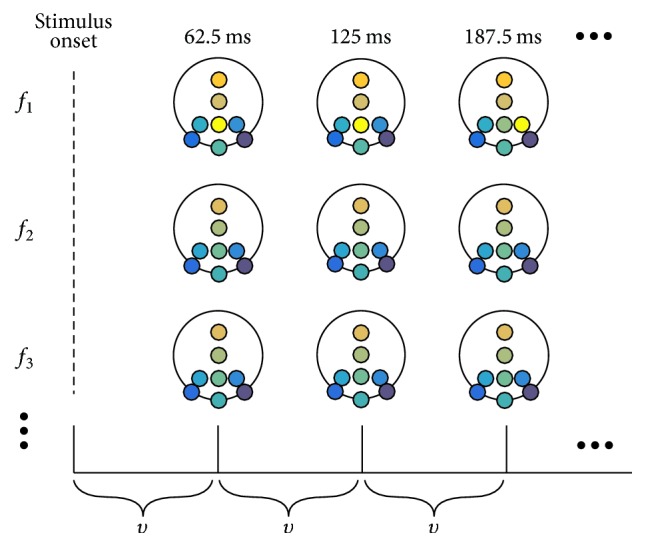
Depiction of how a TFT map is generated. Using the settings for the assessment of cEEG (sampling rate of 256 Hz), the size of each window is *υ* = 16. Hence, each scalp map in the TFT map represents the cluster modes within the cEEG window of size *υ* for each electrode.

To be able to condense the large amount of information obtained from mCPS and make it suitable for visual analysis, we used TFT maps for topographic representation of all *hC* yielded in previous steps. Some previous schemes of Time-Frequency-Topographic visualization can be found in literature [15, 37]. Then, the cEEG section that corresponds to the rEEG segment to be analyzed is windowed, displaying scalp maps with cluster modes of the cEEG windows of size *υ*, which is specified in number of samples (Figure 2). The cluster modes for each channel are assigned only if the mode frequency is greater than threshold *ρ*. For both rEEG and sEEG, *ρ* = 50%; this way, bimodal or multimodal results are avoided.

With regard to the rEEG, it should be mentioned that as the selected runs for analysis with our method were clustered separately, the color labels in a TFT map of ERP condition are the same as a TFT map of no ERP only if it is the same subject and same run; otherwise, this condition may not be satisfied, except for two cases: the color map is bounded between specific RGB values between dark blue and bright yellow, which corresponds with cluster with hexadecimal label “01” (only channel P8 is assigned) and cluster “FF” (all channels are assigned), respectively. Intermediate variations of label color depend on the amount of generated clusters along time.

#### 2.2.5. Intertrial TFT (iTFT) Maps

**Figure 3:**
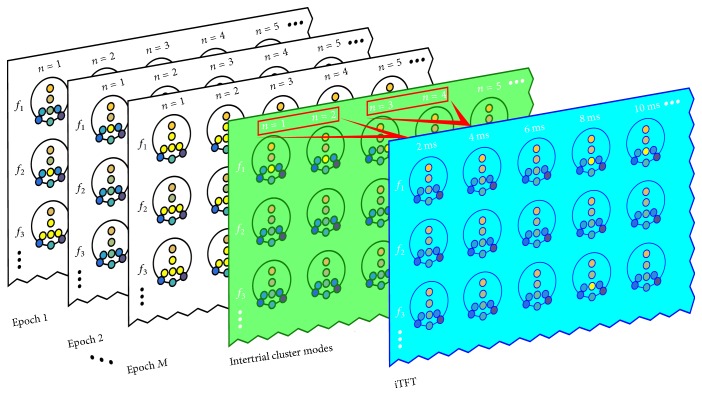
Depiction of how an iTFT map (blue background) is constructed. Following the same principle of a grand average for ERP (computing modes instead), the scalp maps in the green TFT map contain the ITCM of epochs 1,2,…, *M*. For illustration purposes only, let us consider the final step, that is, computing the modes of every window over the discrete time axis, setting *υ* = 2 samples (indicated with the red rectangles and arrows) with a sampling rate of 1 kHz. Thereby, each topographic map in the iTFT map represents the cluster modes of all samples of the array containing the ITCM within the window of size *υ* for each electrode.

An iTFT depicts *hC* modes across epochs. It can be seen as a TFT map containing intertrial cluster modes (ITCM) instead of computing cluster modes over a cEEG segment directly (Figure 3). Regarding the rEEG, for each run of the experimental protocol, the instantaneous phase is computed over the complete run and the clustering is performed before epoch segmentation. After these steps, ERP and no-ERP epochs are taken separately and their ITCM is computed in such a way that the most representative cluster formations over the ERP and no-ERP epochs are retrieved. For the rEEG case, the resultant iTFT map illustrates the most prevalent phase clustering patterns over 1000 ms (duration of trials) with a time window of size *υ* = 16 (62.5 ms).

#### 2.2.6. Levenshtein Distance (LD) and Complementary TF Maps

LD is included to sense relevant differences between ERP and no-ERP epochs. This measure can be defined as the minimum cost of transforming one string into another through a sequence of operations [38]:

(7) LDΦ1,Φ2=min⁡ψTΦ1,Φ2;ψTΦ1,Φ2=∑i=1lψTi,

where Φ_1_ and Φ_2_ are strings constructed with characters *ϕ*_1_, *ϕ*_2_,…, *ϕ*_*z*_ of the same alphabet Γ and **T**_Φ_1_,Φ_2__ = {*T*_1_, *T*_2_,…, *T*_*l*_} represents the set of edit operations to make Φ_1_ = Φ_2_, weighted by function *ψ* ∈ *ℜ*^+^. With *p* → *q* being a simple edit operation and *λ* being the null string, there are three types of transformations: insertions (*λ* → *p*), substitutions (*p* → *q*), and deletions (*q* → *λ*). Adapted to our case, Γ = {“0”, “1”}, *ψ* = 1, and Φ_1_ and Φ_2_ are binary cluster labels of same length; thus the only operation to perform is substitutions of characters. Since clusters labels encipher the membership of electrodes, the maximum LD should be equal to 8 for the extreme case of Φ_1_ = “00000000” (which means that no cluster mode was assigned to any channel due to threshold *ρ*) and a cluster mode with all 8 electrodes within (Φ_2_ = “11111111”).

Furthermore, additional TF maps are generated from the CWT of each channel, which coupled with LD measures, and they help to observe findings in the mCPS information that could be associated with the changes of power due to the ERP over the time series. The LD distances are depicted in Time-Frequency-Levenshtein (TFL) maps.

#### 2.2.7. Framework Pipeline

**Figure 4:**
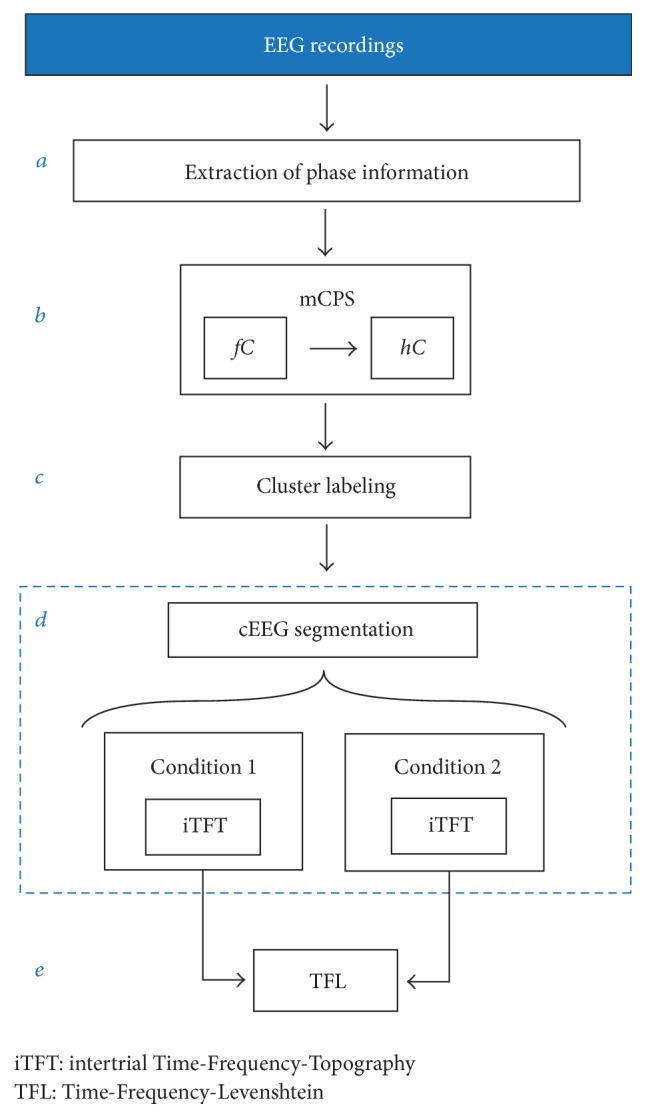
Block diagram of the framework pipeline. Blocks *a*, *b*, and *c* are described in Sections 2.2.1, 2.2.2, and 2.2.3, respectively. This is a general pipeline, and as such another phase extraction technique might be used in block *a* (we opted for CWT). In block *b*, other PS criteria can be introduced (we opted for circular variance) to perform mCPS. The blocks contained in *d* are described in Section 2.2.5, where condition 1 and condition 2 refer to ERP and no-ERP epochs, obtained after segmentation of the time series of cluster labels cEEG. Additionally, TF maps of each channel for both conditions (not depicted in this block diagram) can be used together with the TFL maps of block *e* for visual analysis.

The complete framework pipeline is shown in Figure 4. Once the extraction of phase information of EEG in block *a* and mCPS is performed in block *b*, EEG clusters (cEEG) are labeled in block *c* and then segmented according to the acquisition protocol. For this particular case, condition 1 and condition 2 in Figure 4 correspond to ERP and no-ERP epochs, respectively. Important to notice, segmentation of cEEG occurs after the hexadecimal labeling (block* c*) in order to allow direct comparison between conditions in the iTFT maps, ensuring a one-to-one correspondence among color labels in the topographic scalp layouts of clusters and hexadecimal labels. Finally, the TFL maps (block *e*) highlight dissimilarities over time and frequency of the mCPS outputs for ERP and no ERP.

## 3. Results

**Figure 5:**
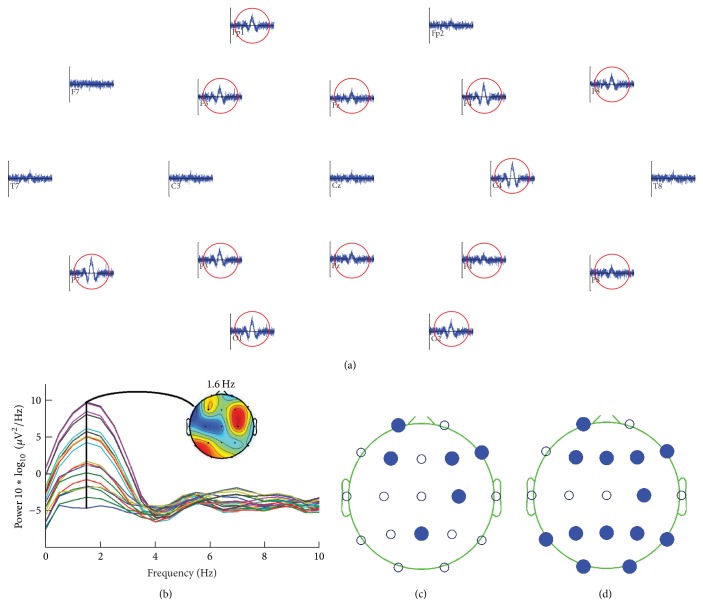
(a) Grand average of each channel; (b) corresponding spectra and scalp distribution of power at 1.6 Hz. Both images were generated with EEGLAB [31]. (c) TFT map at 1500 ms, 1.6 Hz, and SNR = 0.328 dB. (d) TFT map at same time and frequency of grand average, with SNR = 3.16 dB.

Figure 5 summarizes the most remarkable outcome of the experiments with sEEG.  Figure 5(a) shows the grand average of each channel, and Figure 5(b) displays the corresponding spectra of all channels as well as the scalp distribution of power at center frequency *f* = 1.6 Hz.  Figure 5(c) shows a single scalp map extracted from the correspondent TFT maps after applying mCPS over a single trial of sEEG, positioned at 1500 ms (which is where the peak amplitude of the VEP is found) and centered at *f*, with a signal-to-noise ratio (SNR) of 0.328 dB.  Figure 5(d) also shows a single scalp map, at same latency and center frequency *f*, coming from a TFT map generated after applying mCPS over the grand average of the 30 epochs, with SNR = 3.16 dB. By visual inspection, it can be observed in Figure 5(d) that electrodes in blue cluster correspond to those in Figure 5(a), where the VEP is more evident (marked with red circles); it also largely coincides with the scalp areas with highest power at *f* (Figure 5(b)). Remarkable to say, despite the lower SNR in a single trial compared to scalp map of Figure 5(d), mCPS is able to retrieve some of the electrodes within the blue cluster (Figure 5(c)).

**Figure 6:**
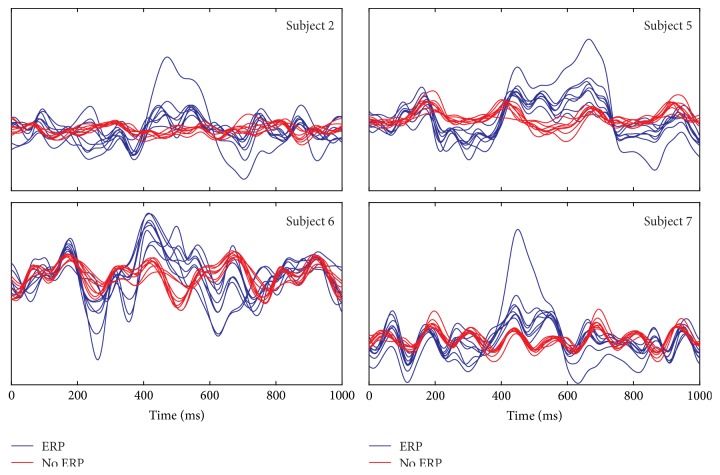
Grand averages of ERP (blue) versus no-ERP (red) condition for each subject (run 4 for S2 and S5, run 7 for S6, and run 6 for S7).

With respect to rEEG, the main attention was on the intertrial analysis searching for differences between ERP and no-ERP conditions, using iTFT maps. Different values of threshold *r* were tested between 0.90 and 0.99 for cluster mode assignments, while *ρ* was fixed at 50% and *υ* = 16 samples. In relation to the data, from the seven runs of each subject, only the testing runs (4–7) were processed with our framework, each of them individually. For reasons of space, only some relevant portions of maps per subject are presented in figures: run 4 for S2 and for S5 (*r* = 0.90 and 0.975, resp.), run 7 for S6, and run 6 for S7 (both with *r* = 0.96).  Figure 6 shows grand averages of all channels for these runs for each subject, contrasting ERP condition (blue) versus no-ERP condition (red). Respecting the TFL and TF maps, only the most illustrating channel is depicted. For the full maps of the runs mentioned before, please refer to http://itzamna.uam.mx/lini/mcps.html.

**Figure 7:**
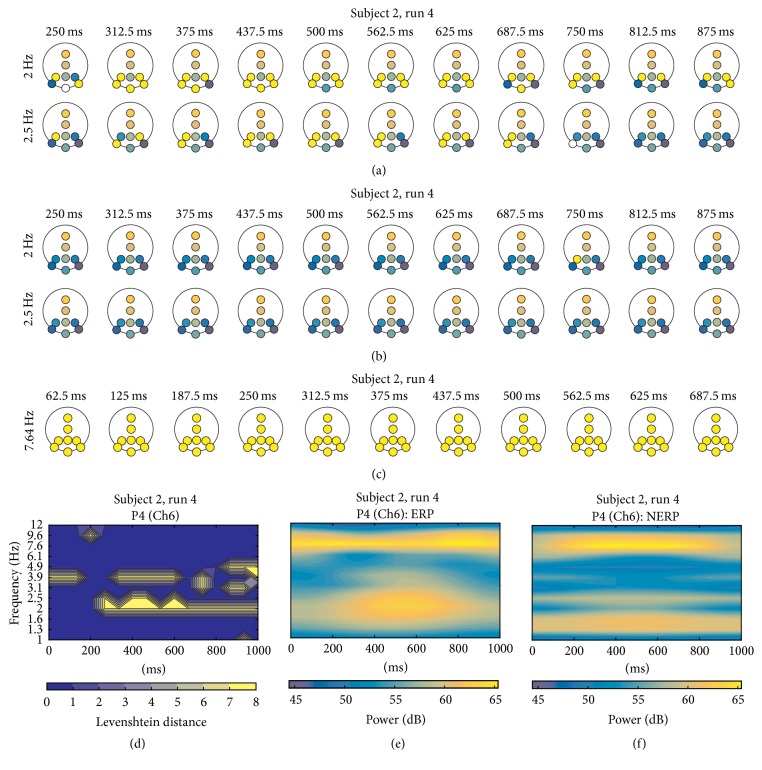
(a) A portion of iTFT maps for S2, showing only the row of bins centered at 2 Hz and 2.5 Hz, from 250 ms to 875 ms of ERP epochs; (b) same *f*_*k*_ depicted for no-ERP epochs. (c) An example of the cluster related with the steady-state artifact. (d) TFL map for P4. (e) TF map for P4 for ERP and (f) TF map for P4 for no ERP.

Results of run 4 for S2 are displayed in Figure 7. In the ERP iTFT map (Figure 7(a)), formations of cluster modes with label “FF” (bright yellow) containing P3-P4-PO7-Oz-PO8 can be observed from 312.5 ms to 750 ms at 2 Hz. The same situation occurs at 2.5 Hz with P3-PO7-P4. No characteristic cluster formation is shown in the no-ERP iTFT map (Figure 7(b)). Noteworthy, run 5 portrayed similar conditions compared to run 4, except that relevant cluster formations were found in bins centered at 1.3, 1.6, and 2 Hz. As for runs 6 and 7, neither ERP nor no-ERP iTFT maps of S2 revealed any characteristic cluster formation. In Figure 7(d), the TFL map for P4 is displayed. Important to highlight, this map depicts yellow areas that coincide (at least visually) with the concentration of power of the P300 wave (Figure 7(e)), particularly for P3, P4, PO7, and Oz (TFL maps for P3, PO7, and Oz can be observed in the complete study). It is also coincidental with the cluster formations described previously in the ERP iTFT map and with the P300 power time course, around 312 ms and 750 ms approximately (Figure 7(e)), which is not the case if such cluster arrangements are compared with no-ERP TF maps (Figure 7(f)).

### 3.1. Steady-State Visual Evoked Potential (SSVEP) Artifact

As depicted in Figure 7(c), it can be observed that cluster “FF” contains all EEG channels at 7.6 Hz over the entire row. This is highly likely to be related to an SSVEP artifact derived from a fixed value of the interstimulus duration (125 ms). This pattern appears in all subjects, with some minor variations of *f*_*k*_. For example, for S2, this fact can be related to the concentration of power around 7.6 Hz in the entire epoch in all TF maps of each channel (Figures 7(e) and 7(f)). This can be verified with almost all TF maps presented for both ERP and no ERP for all subjects.

**Figure 8:**
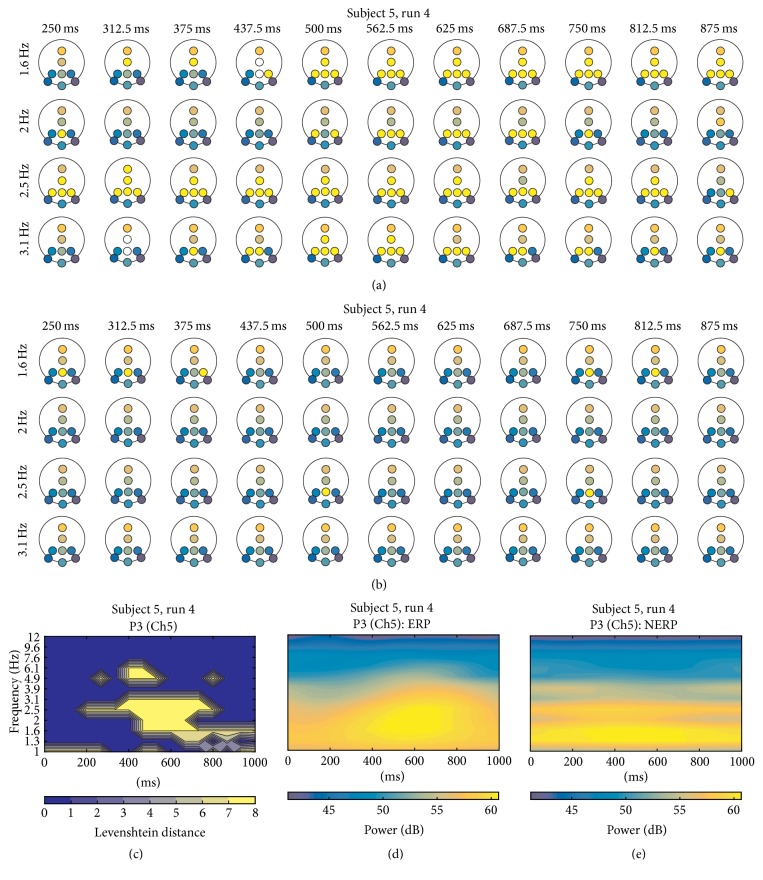
(a) A portion of iTFT maps for S5, showing only the row of bins centered at 1.6, 2, 2.5, and 3.1 Hz, from 250 ms to 875 ms of ERP epochs; (b) same *f*_*k*_ depicted for no-ERP epochs. (c) TFL map for P3. (d) TF map for P3 for ERP, and (e) TF map for P3 for no ERP.

Regarding S5, observations within runs are very similar (*r* = 0.975). For ERP condition (Figure 8(a)), cluster formations of “FF” with parietal channels and Cz clearly coincide with yellow areas of TFL map of P3 (Figure 8(c)) and power concentration of P300 in the TF map (Figure 8(d)). This can be observed over the ERP iTFT cluster formations in bins centered at 1.6, 2, 2.5, and 3.1 Hz (Figure 8(a)). iTFT maps of no ERP (Figure 8(b)) did not show any relevant cluster formation.

**Figure 9:**
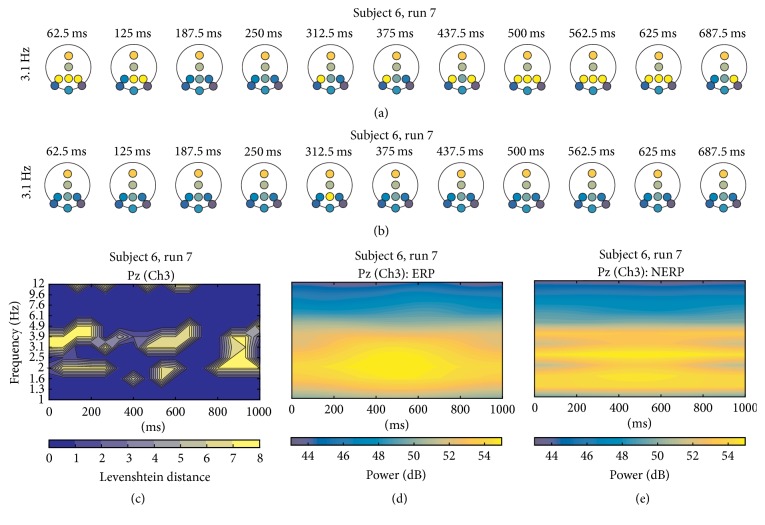
(a) A portion of iTFT maps for S6, showing only the row of bin centered at 3.1 Hz, from 62.5 ms to 687.5 ms of ERP epochs; (b) same *f*_*k*_ depicted for no-ERP epochs. (c) TFL map for Pz. (d) TF map for Pz for ERP and (e) TF map for Pz for no ERP.

For S6 (*r* = 0.96), Figures 9(a) and 9(b) illustrate a section of the correspondent iTFT map of ERP and no-ERP conditions, respectively. The “FF” cluster formations can be observed in the 3.1 Hz bin, which takes place at different time windows. There are no relevant cluster formations over no-ERP map at the same times. The TFL map confirms these differences with the yellow areas for Pz (Figure 9(c)). In this case, the relationship with the power in TF map of P300 wave (Figures 9(d) and 9(e)) is not so evident.

**Figure 10:**
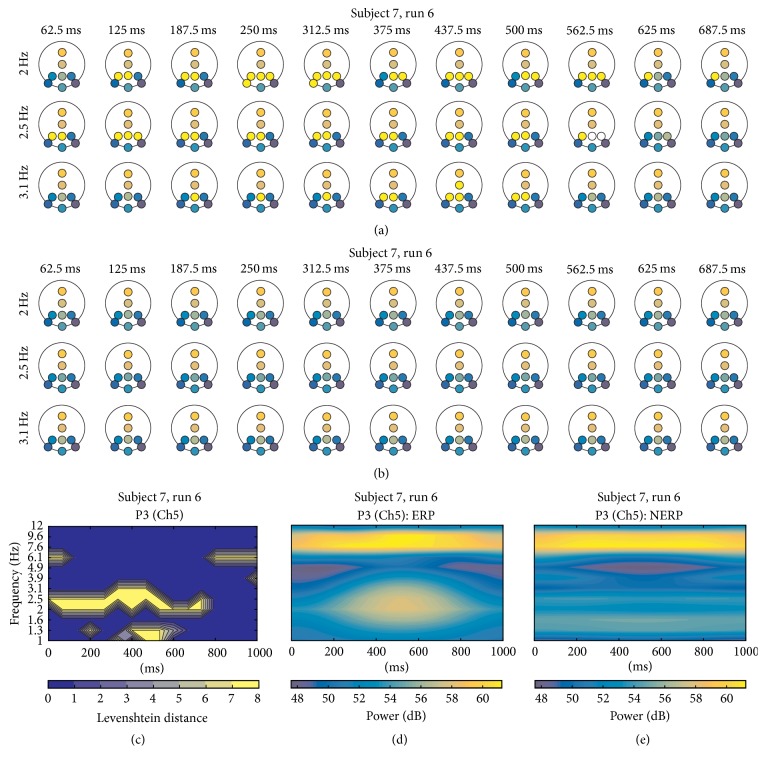
(a) A portion of iTFT maps for S7, showing only the row of bin centered at 2, 2.5, and 3.1 Hz, from 62.5 ms to 687.5 ms of ERP epochs; (b) same *f*_*k*_ depicted for no-ERP epochs. (c) TFL map for P3. (d) TF map for P3 for ERP and (e) TF map for P3 for no ERP.

Concerning S7, in run 6 with a threshold of *r* = 0.96, parietal electrodes stand out again portraying diverse “FF” cluster arrangements over the scalp, mainly at 2, 2.5, and 3.1 Hz (Figure 10(a)), concurring with yellow areas in corresponding TFL map of P3 (Figure 10(c)) and with the power of P300 wave in the TF map (Figure 10(d)). In run 7, the appearance of other cluster formations besides “FF” (perceived in other runs and subjects) was noticeable, with parietal electrodes between 375 and 625 ms.

## 4. Discussion

The findings over the TFT maps of sEEG served as a starting point for leading the research to the analysis with real data, as coincidences of the generated cluster in the single trial and the one over the grand average reflected the ability of mCPS to retrieve the PS information of interest. For rEEG, the iTFT maps exposed several differences between ERP and no ERP, and maybe the most notable and consistent feature was the arrangements of clusters labeled “FF” systematically appearing in ERP maps (Figures 7(a), 8(a), 9(a), and 10(a)) derived from the ITCM computation, contrasting with the absence of such patterns in the no-ERP maps (Figures 7(b), 8(b), 9(b), and 10(b)). This fact is evidenced with the TFL maps (Figures 7(d), 8(c), 9(c), and 10(c)), highlighting the areas of the TF plane with perceptible differences among ERP and no ERP. Such differences can be noted by contrasting Figures 7(e), 7(f), 8(d), 8(e), 9(d), 9(e), 10(d), and 10(e), respectively. Moreover, most of the “FF” appearances can be related (at least by visual inspection) to the P300 wave, given the times and bandwidths where these clusters appear, as most of them were localized within delta and theta ranges, which is consistent with frequency content of a P300 ERP [39–41]. The frequency content of no-ERP epochs observed in Figures 7(f), 8(e), 9(e), and 10(e) could hardly be explained by any neurophysiological event of relevance, but rather it could be due to subharmonics of the SSVEP artifact, as the power concentration can be perceived as extended “lines” throughout the time series.

There were cases (like S6) where analysis with TFL and TF maps did not yield any clear distinction between ERP and no ERP, like run 6, where cluster formations were sporadic and intermittent, making it difficult to establish a relationship with the P300 wave. Noteworthy, samples of ERP and no-ERP epochs are highly unbalanced (each run per subject contains 100 ERP epochs and 500 no-ERP epochs), which reinforces our results distinguishing these conditions, considering the fact that we are using mode as statistical measure, and despite a greater amount of samples of no-ERP epochs, no relevant cluster modes formations were detected.

Another important aspect is related to frequency locking and tracking of frequency flows [42, 43]. A limitation in some methods relying on a narrow band TF decomposition, such as the frequency bins generated with wavelets, is the assumption of frequency stationarity of PS, hiding or masking periods of continuous PS with transient variability of synchronization frequency through time. However, the TFT maps can capture this frequency flow of PS, as it can be observed how the “FF” cluster patterns appear over different low frequency narrow bands, such as *f*_*k*_ = 1.6, 2, 2.5, and 3.1 Hz for S5 (Figure 8(a)) and *f*_*k*_ = 2, 2.5, and 3.1 Hz for S7 (Figure 10(a)).

With respect to hyperparameters, further analysis should be made varying threshold values (*r* and *υ*) in order to evaluate the produced effect in cluster generation and visualization. As mentioned before, several tests were made with different values of *r*, yet the results shown in this work are only for one *r* per subject, which was heuristically selected by identifying the TFL maps that yield a better differentiation of ERP and no-ERP conditions. The method is highly sensitive to *r* variations, and future work can be directed to automate selection of optimal values for *r*.

Even though in these results our framework serves in identifying PS dynamics related to the neural activity of interest organized and structured in clusters of EEG channels, there is still a lot of room for improvement. At this point, our method describes near-zero phase lag relationships between EEG channels (*r* > 0.90 in most of the cases). By definition, volume conduction requires zero phase lag, but a phase difference close to zero is not necessarily due to volume conduction, as this kind of phase associations can be found widespread over the cerebral cortex because of corticothalamic projections [44]. There are some measures such as Phase Lag Index (PLI) [45] or imaginary coherence [46] which deal with volume conduction by discarding zero phase lags, but at the same time these approaches are insensitive to true near-zero phase lag interactions [47].

On the other hand, volume conduction can be addressed by measuring phase reset, which can be detected when a phase shift takes place between two phase-locked signals [48]. This idea can be extended in our framework, trying to find phase resets between EEG channels. Adding other phase differences or phase-locking measures could retrieve different clustering patterns, which along with our already implemented mCPS measure and detection of phase resets could deliver complementary and relevant information.

## 5. Conclusions

Our framework provides a feasible way to address both single and intertrial PS analysis of multivariate neural time series, characterizing the PS variability through time. The majority of PS measures so far suggested in literature such as PLV or PCC are calculated between two signals [6–8] or provide only a global index of synchronization in the case of multivariate measures [19, 20, 23, 27]. Our framework is an alternative for studying the behavior of phase synchronization between all EEG channels at once in a given time window within different bandwidths of interest. Noticeable to say, the framework is not limited to any particular phase extraction technique (further discussion about the selection of these techniques is beyond the scope of this article) and can also easily be adapted to other PS measures like phase coherence, obtaining clusters of phase differences consistency from mCPS. It remains to assess and compare the proposed algorithm to other clustering algorithms in terms of efficiency and computational complexity.

The insight given by the iTFT maps provides a qualitative measure of intertrial cluster consistency, which when combined with the TFL and TF maps becomes helpful to assess which clusters patterns are related to a specific mental task. It should be mentioned that some yellow areas depicted in TFL maps that do not match with the power increase of the P300 wave shown in TF maps could be due to artifacts artificially derived from LD computation or due to other relevant neural information not related to ERP. Further analysis should be made regarding this issue.

Although in this first approach mCPS was applied over synthetic signals and P300 wave data with relatively few electrodes, the aim of this work was merely to illustrate the framework pipeline and how it describes PS patterns. As mentioned before, our work attempts to encompass a broader variety of cognitive states. For example, in the context of BCI, our framework might be useful for the characterization of mental tasks suitable for endogenous BCI paradigms with no external stimuli in the system. Then, feature extraction could be performed from mCPS outcome for asynchronous (self-paced) BCI classification, distinguishing idle state from a specific mental task. Additionally, when exploring higher density EEG (64 channels or more), this framework could be used as a channel optimization tool finding the clusters of electrodes that contribute the most to characterization of a mental state.

Electrical signals from brain sources are volume conducted through nervous tissue, cerebrospinal fluid, skull, and scalp. Hence, an underlying issue in EEG recordings regards the single source contamination of multiple sensors via volume conduction. The EEG recorded over the scalp does not necessarily capture the direct activity underneath the electrode but a weighted mixture of different sources (neural or artifact). Then, distinction between volume conduction and true synchrony remains an open issue. Some authors have reported that methods for improving spatial resolution of EEG, such as scalp current density profiles (SCD), seem convenient as preprocessing steps before the estimation of PS [7, 8]. For future work, it should be interesting to study the effects of rereferencing. Again, in the BCI field, it could be assessed if rereferencing enhances performance using phase clusters as features for classification, bearing in mind the fact that the original phase delays may be distorted. It should be pointed out that no additional preprocessing was made, preserving the data as raw as possible. Further approaches for addressing volume conduction should be considered in forthcoming research.

Finally, to summarize the contributions, the proposed framework incorporates several features useful for PS analysis, such as iTFT and TFL maps, taking into account some aspects like frequency nonstationarity and flexibility of use of other synchronization measures besides PLF. The LD is applied as a metric for better distinction of differences between conditions, highlighting synchrony differences between ERP and no-ERP epochs, mainly at delta and theta bands. Additional information like the steady-state artifacts at 7.6 Hz is also retrieved and depicted in iTFT maps. Taking EEG as the view port of cortical activity, our framework provides a new insight into terms of large-scale integration of emerging synchrony patterns of instantaneous phase during cognitive tasks, depicted in phase-related cluster arrangements over the time series of EEG signals.

## Figures and Tables

**Algorithm 1:**
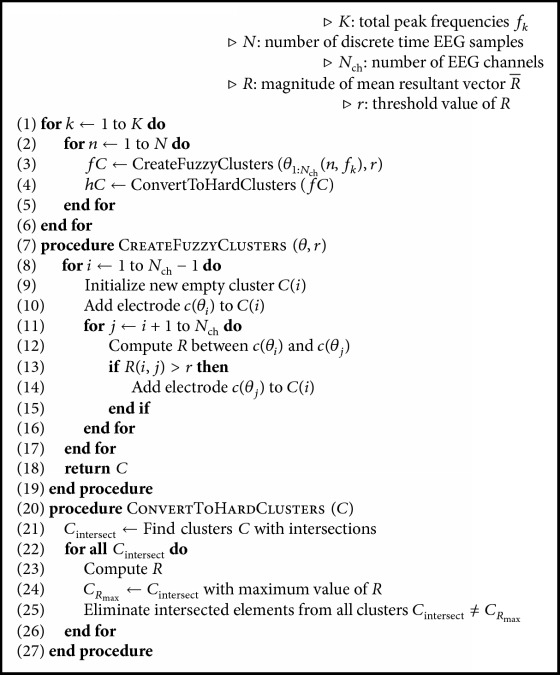
Multivariate Time Series Clustering by Phase Synchrony (mCPS).
